# Insights into mechanisms of graft-versus-host disease through humanised mouse models

**DOI:** 10.1042/BSR20211986

**Published:** 2022-09-05

**Authors:** Amal Elhage, Chloe Sligar, Peter Cuthbertson, Debbie Watson, Ronald Sluyter

**Affiliations:** 1 Illawarra Health and Medical Research Institute, Wollongong NSW 2522, Australia; 2 Molecular Horizons and School of Chemistry and Molecular Bioscience, University of Wollongong, Wollongong NSW 2522, Australia

**Keywords:** Allogeneic haematopoietic stem cell transplantation, Antigen presenting cell, Graft-versus-host disease, Inflammation, Lymphocyte, Xenogeneic graft-versus-host disease

## Abstract

Graft-versus-host disease (GVHD) is a major complication that occurs following allogeneic haematopoietic stem cell transplantation (HSCT) for the treatment of haematological cancers and other blood-related disorders. GVHD is an inflammatory disorder, where the transplanted donor immune cells can mediate an immune response against the recipient and attack host tissues. Despite over 60 years of research, broad-range immune suppression is still used to prevent or treat GVHD, leading to an increased risk of cancer relapse and infection. Therefore, further insights into the disease mechanisms and development of predictive and prognostic biomarkers are key to improving outcomes and reducing GVHD development following allogeneic HSCT. An important preclinical tool to examine the pathophysiology of GVHD and to understand the key mechanisms that lead to GVHD development are preclinical humanised mouse models. Such models of GVHD are now well-established and can provide valuable insights into disease development. This review will focus on models where human peripheral blood mononuclear cells are injected into immune-deficient non-obese diabetic (NOD)-*scid-*interleukin-2(IL-2)Rγ mutant (NOD-*scid*-IL2Rγ^null^) mice. Humanised mouse models of GVHD can mimic the clinical setting for GVHD development, with disease progression and tissues impacted like that observed in humans. This review will highlight key findings from preclinical humanised mouse models regarding the role of donor human immune cells, the function of cytokines and cell signalling molecules and their impact on specific target tissues and GVHD development. Further, specific therapeutic strategies tested in these preclinical models reveal key molecular pathways important in reducing the burden of GVHD following allogeneic HSCT.

## Introduction

Graft-versus-host disease (GVHD) is an inflammatory disorder that can occur following allogeneic haematopoietic stem cell transplantation (HSCT) [1,2]. Allogeneic HSCT is an efficient and cost-effective treatment for life-threatening blood or bone marrow related disorders in adults and children, which can include haematological malignancies, bone marrow failure, haemoglobinopathies and immunodeficiencies [3]. It is estimated that over 700,000 allogeneic HSCTs have been performed to date, with over half being performed in people with acute myeloid leukaemia, and the rest performed in those with acute lymphoblastic leukaemia, myelodysplastic syndrome/myeloproliferative neoplasms, chronic myeloid leukaemia, plasma cell disorders, lymphomas or non-malignant disorders [4].

Allogeneic HSCT is a curative therapy for haematological malignancies and other blood-related disorders. This therapy aims to regenerate the immune system following pre-conditioning [5]. In addition, allogeneic HSCT mediates a beneficial graft-versus-tumour (GVT) response in recipients with a malignancy, which can eliminate residual cancer cells and prevent disease relapse [6,7]. However, GVHD is a major complication post-transplantation with 30–70% of recipients developing some form of this disease, being fatal in up to 30% of cases [8]. Compounding this, current treatments used to reduce or treat GVHD, including broad range immunosuppression, can lead to cancer relapse and infection, and along with GVHD, remain major causes of morbidity and mortality in allogeneic HSCT recipients [5]. Therefore, new therapies are required to reduce GVHD and improve outcomes for people following allogeneic HSCT.

During GVHD donor immune cells recognise the recipient tissues as foreign and mount an immune mediated attack on host tissues, leading to tissue damage and organ failure [1]. GVHD is considered a T cell-mediated disease; however, specific T cell subsets, such as T helper (Th) 1, Th17, regulatory T cells (Tregs) and other immune cells such as B cells and natural killer (NK) cells, can also play a role in GVHD development [9]. Further, cell signalling molecules, especially cytokines [10,11] including chemokines [12], mediate T-cell activation and GVHD development.

While several factors may contribute to the severity of GVHD in the recipient, including the level of human leukocyte antigen (HLA) mismatch, pre-conditioning regimes, and the source of donor cells [8], further research is required to better understand the mechanisms of GVHD pathophysiology. GVHD development can be classified as acute or chronic, with each form displaying unique immunological processes [13]. GVHD causes damage to the skin, liver, gastrointestinal tract and lungs [1,14]. However, clinically GVHD can manifest differently in recipients, with different time courses for disease progression, and different tissues impacted [14] and this heterogeneity makes GVHD difficult to treat. Despite over 60 years of research, GVHD remains a major complication of allogeneic HSCT, and further research into this debilitating disease is vital.

Mechanisms for GVHD progression and the development of treatments for the prevention of this disease have come mainly from allogeneic mouse models, which do not always translate to the clinical setting [15]. Humanised mouse models of GVHD, where human peripheral blood mononuclear cells (hPBMCs) are injected into immune deficient mice, may offer an advantage as they examine the human immune response, which may more readily translate to the clinic [16]. However, human immune cell engraftment is often limited in these models. Despite T cells readily engrafting, B cells and myeloid cells are not typically present in humanised mice [17]. Furthermore, humanised mouse models are heterogeneous and not all tissues will be affected. This may depend on the pre-conditioning regime or source of human cells, with minimal gastrointestinal tract GVHD observed in non-irradiated mice [18]. Despite this, humanised mouse models do afford a valuable tool to provide insight into mechanisms of disease and key molecular and cellular targets important in GVHD development following allogeneic HSCT.

This article will briefly review current approaches in allogeneic HSCT, before discussing GVHD and humanised mouse models. Finally, this article will detail cellular and molecular mechanisms in GVHD as observed through studies in humanised mouse models using functionally related strains.

## Allogeneic haematopoietic stem cell transplantation

Allogeneic HSCT is largely used to treat haematological malignancies [3]. This treatment involves the transplantation of haematopoietic stem cells (HSCs) from a fully or partially matched healthy donor to a pre-conditioned recipient. Pre-conditioning involves either a myeloablative or reduced intensity conditioning regime, which are used at similar frequencies clinically [8], with the latter allowing the transplantation of higher risk patients [19]. HLA-matched sibling donors remain the transplant donor of choice, with HLA-matched unrelated donors preferred in the absence of matched sibling donors and together comprise two-thirds of allogeneic HSC transplants in equal frequency. In the absence of either of these two donor types, HLA-mismatched unrelated donors, haploidentical donors (that is a sibling or other relative who shares exactly one HLA haplotype with the recipient), and unrelated umbilical cord donors are used [8]. Peripheral blood serves as the main source of HSCs, being used in 85% of allogeneic HSC transplants, with HSCs obtained from bone marrow, both peripheral blood and bone marrow, or cord blood used in the remainder of transplants [8]. Ease of collection and faster recovery of donors, and improvements in engraftment and disease-free and overall survival in patients with high risk haematologic malignancies have contributed to the widespread use of peripheral blood HSCs [20].

Despite the life-saving benefits of allogeneic HSCT in people with haematologic malignancies, GVHD occurs in 30–70% of recipients [8]. To manage this risk, prophylaxis is used. Current prophylaxis against GVHD commonly includes broad range immunosuppression with a calcineurin inhibitor, cyclosporin (CsA) or tacrolimus, with methotrexate or another anti-metabolite [21]. The depleting agent rabbit anti-thymocyte globulin or anti-T cell globulin (ATG) is used in recipients at high risk of GVHD, with either commonly used for non-manipulated transplants, and ATG typically used for T cell-depleted transplants [22]. Cyclophosphamide is an alkylating agent and post-transplant cyclophosphamide (PTCy) is commonly used in haploidentical transplants [23], with increasing support for its use as a combined therapy in matched related or unrelated transplants [24,25].

In the event of GVHD development, treatment of patients is dependent on GVHD type. First-line treatment of acute GVHD is the use of corticosteroids, including systemic methylprednisolone, with lower doses of methylprednisolone or prednisolone used in isolated skin or upper gastrointestinal tract GVHD and topical steroids used for skin GVHD [22]. Treatment of steroid-resistant GVHD, however, is an ongoing area of controversy and research, with second-line options comprising anti-metabolites including methotrexate and pentostatin, depletion agents including alemtuzumab (CD52^+^ cell depletion), anti-proliferative reagents including mycophenolate mofetil or sirolimus (rapamycin), targeting activation with basiliximab [interleukin (IL)-2 receptor (IL-2R) blockade] or daclizumab (IL-2R blockade), using protease inhibitors like α1-antitrypsin or Janus-associated kinase (JAK) inhibitors, targeting tissue homing molecules including vedolizumab [alpha 4 β 7 (α4β7) integrin blockade] or using extra-corporal photopheresis or faecal microbiota transplantation [22].

First-line treatment of chronic GVHD remains corticosteroids [21], with second-line options including targeting activation with calcineurin inhibitors, anti-proliferative reagents including mycophenolate mofetil, mammalian target of rapamycin (mTOR) inhibitors, adenosine analogues including pentostatin, proteasome inhibitors, tyrosine kinase inhibitors including ibrutinib or extra-corporal photopheresis [22,26].

To meet this growing need to improve patient outcomes following allogeneic HSCT different approaches and new combinations of the above GVHD therapies continue to be investigated. Moreover, research continues to investigate a broad range of new therapeutics including, but not limited to, co-stimulatory blockade with abatacept (CD28 blockade), targeting cytokines using etanercept [tumour necrosis factor (TNF-α) blockade] and tocilizumab (IL-6R blockade), use of the kinase inhibitor ruxolitinib (JAK 1/2 inhibition) or the anti-proliferative reagent azacitidine (DNA-methyltransferase inhibition), glycoprotein targeting with sitagliptin (CD26/dipeptidyl peptidase-4 inhibition), and regulatory therapies including low dose IL-2 and transfer of Tregs [27].

Use of each new GVHD therapy or combination therapy needs to balance the risk of GVHD prevention with the risk of engraftment failure, infection and in the case of haematological malignancies, disease relapse. To this end humanised mice have provided valuable models in understanding the mechanisms of GVHD, as well as in the testing the efficacy of new GVHD therapies and their impact on GVT immunity.

## Graft-versus-host disease

GVHD is still a major issue in allogeneic HSCT recipients, despite the numerous treatment options outlined above, with 30–50% of recipients developing acute GVHD [28] and 30–70% developing chronic GVHD [29]. Acute and chronic GVHD were originally defined by the time at which they occur, before or after 100 days post-transplantation, respectively [30], but they are now defined by differences in pathology [13], and are recognised to occur outside of those original timeframes and to overlap in some patients [31]. Currently, acute GVHD occurring before and after 100 days is defined as classic and late acute GVHD, respectively, and chronic GVHD in the absence or presence of acute GVHD defined as classic and overlap chronic GVHD, respectively, irrespective of any time limit [31].

Acute GVHD is mediated by host antigen-presenting cells (APCs) and donor T cells in a three-stage process, defined as the afferent, efferent and effector phases [32]. The afferent phase is initiated when pre-transplant conditioning or allogeneic HSCT causes early tissue damage [33], which leads to the release of: damage-associated molecule patterns (DAMPs), such as adenosine 5′-triphosphate (ATP) [34]; pathogen-associated molecule patterns (PAMPs), such as lipopolysaccharide [35]; and pro-inflammatory cytokines such as TNF-α and IL-6 [36]. Together these molecules activate host APCs, which initiate the second phase of GVHD [37]. Notably, lower intensity pre-conditioning regimes, which lessen initial tissue damage, minimise APC activation and result in reduced GVHD [38,39].

The efferent phase begins when activated host APCs stimulate donor T cells. Donor CD4^+^ and CD8^+^ T cells, respectively, recognise host major histocompatibility complex (MHC) class II and I molecules on APCs, with mismatches between host and donor MHC molecules driving the activation of T cells [40]. Moreover, in instances where donor and host MHC molecules are matched, differences in minor histocompatibility antigens between the host and donor may result in T-cell activation [41]. Following stimulation and activation, donor T cells proliferate and differentiate into a number of effector T-cell subsets [9]. For example, CD4^+^ T cells can differentiate into Th1 and Th17 subtypes following T-cell receptor (TCR) stimulation and co-stimulation with cytokines like IL-12 [42] or transforming growth factor (TGF)-β, IL-6 and IL-23 [43], respectively. Similarly, TCR and cytokine co-stimulation during GVHD directs the proliferation and differentiation of donor CD8^+^ T cells into various subsets with differing cytokine profiles [44]. Regulatory cells, including Tregs and myeloid-derived suppressor cells (MDSCs), have the ability to reduce T-cell activation and prevent effector T cells from damaging host tissues. The mechanisms involved in Treg- and MDSC-mediated suppression in GVHD have been reviewed in detail elsewhere [45,46]. Briefly, Tregs suppress GVHD through a variety of mechanisms involving direct cytolysis of effector T cells, secretion of anti-inflammatory cytokines and direct interaction with dendritic cells (DCs) [46]. While MDSCs can suppress GVHD directly by the release of anti-inflammatory cytokines and indirectly by promoting the induction and expansion of Tregs [45].

The effector phase occurs when activated alloreactive donor T cells migrate to host tissues, including the gastrointestinal tract, skin, liver and lung, and initiate a severe inflammatory response [33]. T-cell migration is influenced by the cell surface expression of homing molecules, including cutaneous lymphocyte antigen [47] and α4β7 integrin [48], which interact with addressins on host cells [49]. Following migration to host tissues, activated donor T cells release pro-inflammatory cytokines in a ‘cytokine storm’ [33] and directly attack host cells through a number of mechanisms. Th1 and Th17 cells produce the pro-inflammatory cytokines interferon (IFN)-γ [50] and IL-17 [51], respectively, to promote GVHD. IFN-γ makes host cells in the gastrointestinal tract and skin more susceptible to GVHD damage [52,53] while IL-17 increases the production of other cytokines including IL-6 and IFN-γ [54]. Additional to this ‘cytokine storm’, alloreactive CD4^+^ and CD8^+^ T cells directly attack target host cells through the Fas/FasL pathway and perforin/granzyme pathways, respectively [33]. Furthermore, there is increasing evidence that non-coding RNAs influence T-cell responses during GVHD [55]. For example, microRNA (miR)142 [56] and miR17-92 [57] increase proliferation and survival of T cells, while miR146a [58] and miR181a/b [59] decrease T-cell proliferation and survival following allogeneic HSCT. Additionally, other miRs have been shown to increase or decrease Th1, Th17, Th2 and Treg differentiation and influence activation and trafficking of cytotoxic T cells [55]. Altogether, this tissue damage and cytokine release leads to the further release of DAMPs and PAMPs, and increased T-cell activation in a positive feedback cycle.

Chronic GVHD has also been described to occur in three phases [31]. The first phase begins with cytotoxic therapy (to treat either the underlying disease or acute GVHD), infection and/or acute GVHD initiating the release of DAMPs, PAMPs and cytokines [60]. These molecules induce the rapid activation of neutrophils, macrophages, DCs, fibroblasts and endothelial cells [61], as well as B cells [62]. The second phase is characterised by the activation of T and B cells by APCs, and thymic injury from the pre-conditioning regime, resulting in a loss of peripheral tolerance [61]. Follicular Th cells [63], Th polarisation [64], antibody responses [65] and B-cell activating factor [66] have all been associated with chronic GVHD in patients. Moreover, many tolerogenic factors are impaired [61] and chronic GVHD patients have reduced regulatory cells, including Tregs [67] and C-X-C chemokine receptor (CXCR) 3 expressing regulatory NK cells [68]. The final phase of chronic GVHD involves localised tissue fibrosis in damaged areas [69]. This fibrosis occurs when macrophages promote fibroblast activation to induce abnormal tissue repair, including the excess production of collagen and biglycan [61].

Despite current prophylaxis and treatment options, GVHD is a prevalent complication following allogeneic HSCT and remains a major cause of morbidity and mortality. A better understanding of the mechanisms of GVHD pathogenesis is needed to overcome this limitation and improve the success of allogeneic HSCT as a treatment for haematological malignancies. To this end, humanised mice have provided valuable models in understanding the mechanisms of GVHD, as well as the testing of the efficacy of new GVHD therapies and their impact on GVT immunity in relation to haematological malignancies. However, such models are limited mainly to acute GVHD, which will be the focus of the remaining article, with acute GVHD denoted simply as GVHD hereafter.

## Humanised mouse models of GVHD

Humanised mouse models can be broadly classified as those that involve the expression of specific human gene products within mice, including instances in which a specific mouse gene is replaced by the human orthologue or those that involve the injection of human cells into immunodeficient mice [17]. The latter of these have been widely used to generate humanised mouse models of GVHD to better understand mechanisms and to test therapeutics in this disease [16,18].

Most commonly, GVHD is studied by the transplantation of hPBMCs into immunodeficient mice encoding targeted mutations in the *Il2rg* gene [70], which results in the complete absence of the IL-2R γ-chain (*Il2rg^tm1Wjl^*) or a truncated IL-2R γ-chain lacking the intracytoplasmic domain (*Il2rg^tm1Sug^*), both of which prevent murine NK cell development [71]. The background of the immunodeficiency in mice is typically the *scid* mutation in the gene encoding the DNA-dependent protein kinase catalytic subunit (*Prkdc^scid^*) or the targeted *tm1Fwa* mutation in the recombination-activating gene 2 (*Rag2^tm1Fwa^*), both of which impair murine T- and B-cell development [71]. Mice are often on a non-obese diabetic (NOD) mouse background, a strain that carries a polymorphism in the gene encoding the signal regulatory protein-α (SIRPα), which enhances murine SIRPα-human CD47 interactions and promotes engraftment of human haematopoietic cells [72]. Mice encoding the above mutations readily engraft hPBMCs and subsequently develop GVHD [70,71].

**Figure 1:**
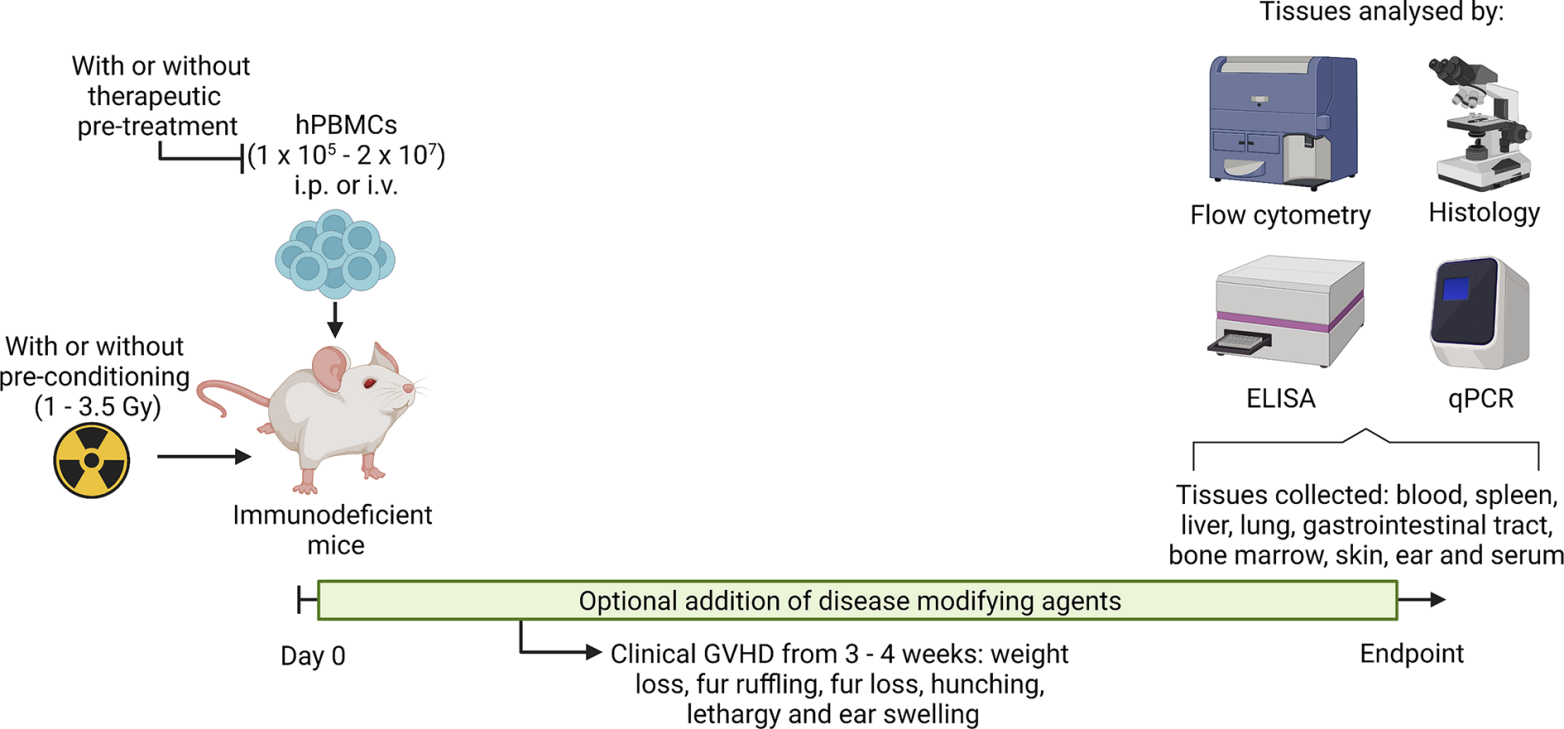
Humanised mouse models of graft-versus-host disease To establish a model of GVHD, non-pre-conditioned or pre-conditioned (1–3.5 Gy radiation) NOD.Cg-*Prkdc^scid^Il2rg^tm1Wjl^* (NSG), NOD.*Cg-Prkdc^scid^Il2rg^tm1Sug^* (NOG) or NOD.*Prkdc^scid^Il2rg^null^* (NPG) mice are injected intraperitoneally (i.p.) or intravenously (i.v.) with 1 × 10^5^ – 2 × 10^7^ hPBMCs (Day 0). Mice begin to develop clinical signs of GVHD from 3 to 4 weeks post-hPBMC injection. Clinical signs of GVHD include weight loss, fur ruffling, fur loss, hunching, lethargy and ear swelling. Disease modifying reagents can be administered at selected time points as desired. At experimental or humane endpoints, tissues such as blood, spleen, liver, lung, gastrointestinal tract, bone marrow, skin, ear and serum can be collected and analysed. Techniques for analysis of tissues include, but are not limited to, flow cytometry, histology (including immunohistochemistry), enzyme-linked immunosorbent assay (ELISA) (and other immunosorbent assays and biochemical assays) and quantitative real-time polymerase chain reaction (qPCR) (and other gene expression assays).

The most commonly used hPBMC mouse model to study GVHD involves three related strains (Figure 1) with NOD.Cg-*Prkdc^scid^Il2rg^tm1Wjl^* (NSG) mice the most published. The first detailed report using this strain revealed that intravenous (i.v.) injection with as few as 5 × 10^6^ hPBMCs into irradiated (2 Gy) NSG mice results in lethal GVHD [73]. This same study also revealed that i.v. injection of 20 × 10^6^ hPBMCs into non-irradiated NSG mice could induce lethal but delayed GVHD. A preliminary observation from the same group indicated that intraperitoneal (i.p.) injection of 20 × 10^6^ hPBMCs into non-irradiated NSG results in human leukocyte engraftment, but details regarding GVHD development in this study were limited [74]. Since these landmark studies, others have used irradiated and non-irradiated NSG mice to establish models of GVHD (Table 1). The second variation of this model uses NOD.Cg-*Prkdc^scid^Il2rg^tm1Sug^* (NOG) mice. The i.v. or i.p. injection of at least 1 × 10^4^ or 1 × 10^7^ hPBMCs, respectively, into irradiated (2.5 Gy) NOG mice results in lethal GVHD (Table 1), with the i.v. injection of 1 × 10^7^ hPBMCs also able to induce lethal GVHD in non-irradiated NOG mice [75]. The third variation of this model uses NOD.*Prkdc^scid^Il2rg^null^* (NPG) mice, with GVHD also able to occur in the absence of irradiation [76] (Table 1), but precise details of this mouse strain are scant, appearing to be NSG mice bred by Beijing Vitalstar Biotechnology.

**Table 1:** Humanised mouse models of graft-versus-host disease

Host (pre-conditioning)	No. of PBMCs (route)	Human cells engrafted	Tissues engrafted	Histological GVHD	Ref.
NSG (2 Gy)	0.5, 1 or 2 × 10^7^ (i.v.)	CD45^+^, CD4^+^ and CD8^+^	Blood, spleen and bone marrow	Spleen, liver, lung and duodenum	[73,74]
NSG (2 Gy)	1 × 10^4^, 1 × 10^5^, 1 × 10^6^ or 1 × 10^7^ (i.v.)	CD45^+^ and CD3^+^	Liver, skin, lung and kidney	Liver, lung, skin and kidney with ≥1 × 10^5^ PBMCs	[87]
NSG (2.5 Gy)	1, 2, 3 or 5 × 10^7^ (i.p.)	CD45^+^, CD3^+^ CD4^+^ and CD8^+^	Blood, spleen, liver, lung and bone marrow	Lung	[88]
NSG	1 × 10^7^ (i.v.)	CD45^+^, CD3^+^, CD4^+^ and CD8^+^, CD4^+^CD45RO^+^CD27^−^	Blood, spleen, bone marrow and lymph node	Not reported	[82]
NSG	1 × 10^7^ (i.p.)	CD45^+^	Blood, spleen and abdominal fluid (ascites)	Not reported	[83]
NSG	1 × 10^7^ (i.p.)	CD45^+^, CD3^+^, CD4^+^ and CD8^+^	Blood, spleen and liver	Liver, lung, skin and ear, but limited intestinal	[84–86]
NOG (2.5 Gy)	5 × 10^6^ (i.v.)	CD45^+^ and CD3^+^	Blood, spleen and bone marrow	Liver, lung and kidney	[75]
NPG	1 × 10^6^ (i.v.)	Not reported	Not reported	Not reported	[76]
NPG (2 Gy)	3 × 10^6^ (i.v.)	CD45^+^ and CD3^+^	Blood, spleen, liver, lung and intestine	Spleen, liver, lung and intestine	[158]

Abbreviations: CD3^+^, T cells; CD4^+^, CD4^+^ T cells; CD8^+^, CD8^+^ T cells; CD4^+^CD45RO^+^CD27^−^, effector CD4^+^ T cells; CD45^+^, leukocytes; GVHD, graft-versus-host disease; i.v., intravenous; i.p., intraperitoneal; NOG, NOD.*Cg-Prkdc^scid^Il2rg^tm1Sug^*; NPG, NOD-*Prkdc^scid^Il2rg^null^*; NSG, NOD.Cg-*Prkdc^scid^Il2rg^tm1Wjl^*; PBMC, peripheral blood mononuclear cell.

The studies above indicate that the pre-conditioning of mice with irradiation and the subsequent tissue damage is not necessary for the induction of GVHD following hPBMC injection. These findings parallel reports in other models, where it was revealed that i.v. injection of human peripheral blood T cells into NOG mice [77] or the retro-orbital injection of human bone marrow-derived mononuclear cells into NOD.Cg-*Kit^W-J^Tyr^+^Prkdc^scid^Il2rg^tm1Wjl^* (NBSGW) mice [78] could induce GVHD in the absence of pre-conditioning. Notably, elevated mouse cytokines and human T cells were present in tissues from non-irradiated NSG mice 9 days post-human T-cell injection [77]. Likewise, T-cell proliferation could be detected as early as 7 days post-human T-cell injection in non-irradiated NBSGW mice, with the proliferation serving as a reliable marker of GVHD development [78]. Human T-cell proliferation can also be detected 6 days post-hPBMC injection in non-irradiated NSG mice [79]. Thus, these studies further suggest that GVHD can be induced shortly after transplantation in the absence of radiation-associated tissue damage. However, the lack of pre-conditioning with irradiation seems to be associated with limited gastrointestinal GVHD in NSG [80] and NBSGW [78] mice, but direct comparisons between irradiated and non-irradiated humanised mice are required to confirm this notion.

**Figure 2:**
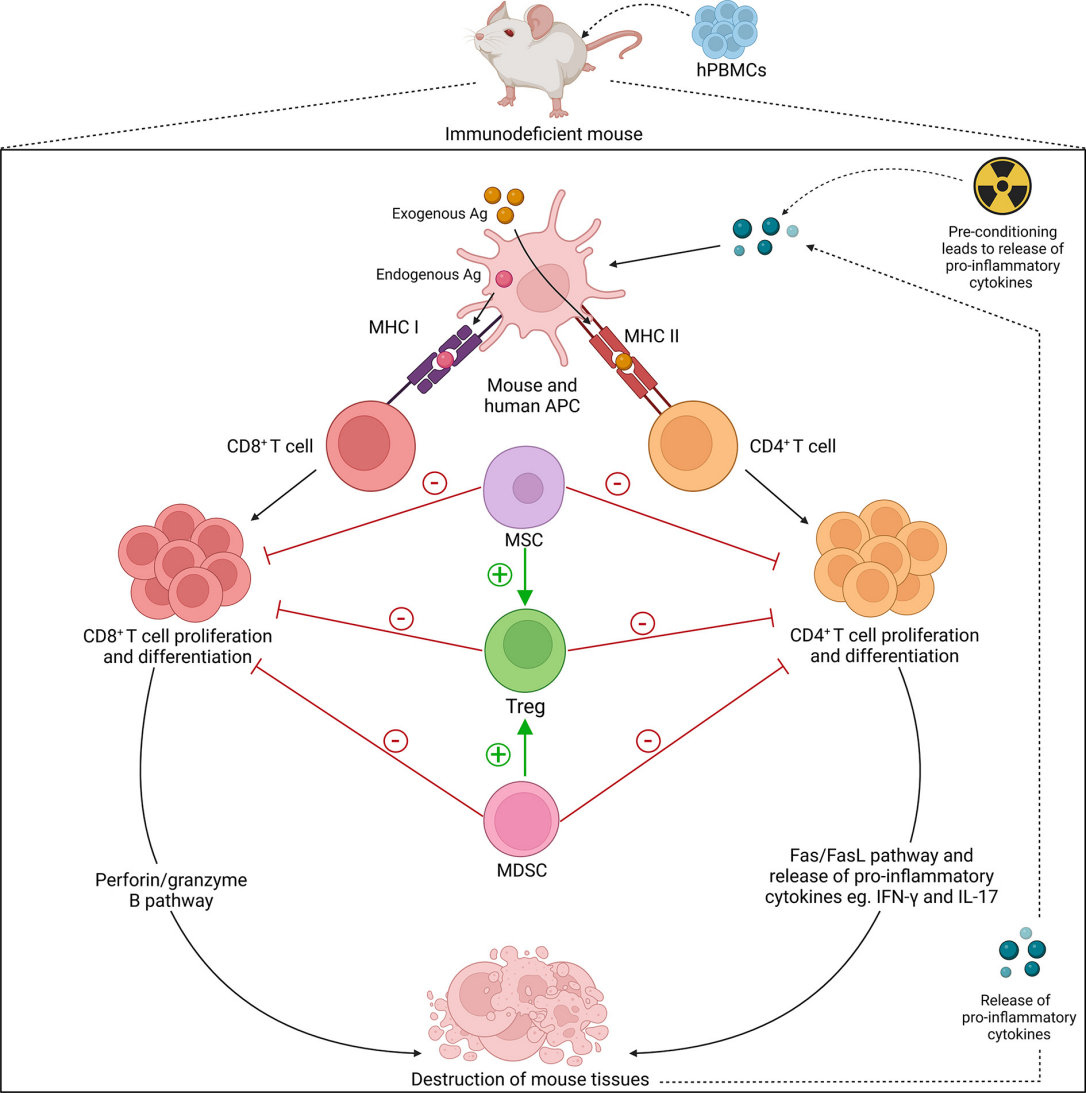
Cellular mechanisms in graft-versus-host disease Immunodeficient mice are humanised with hPBMCs. Both mouse and human APCs become activated through interaction with either endogenous or exogenous antigen (Ag). Pre-conditioning regimens lead to the release of cytokines. CD4^+^ and CD8^+^ T cells are then stimulated by activated mouse or human APCs. CD4^+^ and CD8^+^ T cells proliferate and differentiate to mediate tissue destruction. Allogeneic mouse studies suggest that these processes may involve the Fas/FasL with pro-inflammatory cytokine release and perforin/granzyme B pathways, respectively. Destruction of mouse tissues leads to the further release of cross-reactive pro-inflammatory cytokines, including IFN-γ and IL-17, which creates an inflammatory immune response via a positive feedback loop. Tregs suppress the proliferation of CD4^+^ and CD8^+^ T cells and attenuate GVHD severity. MSCs and MDSCs suppress T-cell differentiation and proliferation whilst simultaneously stimulating the generation of Tregs. APCs may also have tolerogenic roles to suppress GVHD development (not shown).

Finally, it should be noted an alternate hPBMC mouse model of GVHD was originally established by the i.v. injection of 10 × 10^6^ hPBMCs into irradiated C.Cg-*Rag2^tm1Fwa^IL2rγ^tm1Sug^* (BRG) mice (more commonly referred to as BALB/c-*Rag2^−/−^IL2rγ^−/−^* mice) and which also leads to lethal GVHD [81], although at a slower rate than irradiated NSG mice injected with the same number of hPBMCs [82]. Compared with the other three models, this model is used far less frequently and will not be discussed further. Therefore, the following sections will focus on humanised mouse models established by the injection of hPBMCs into NSG, NOG or NPG mice, broadly grouped as NOD-*scid*-IL2Rγ^null^ mice. Use of these humanised mouse models has promoted insights into the mechanisms of GVHD (Figure 2).

## Cell subset mechanisms in GVHD

Cell subset manipulation or transfer experiments in humanised mice have provided understanding of the mechanisms of GVHD development by examining the role of specific effector T-cell subsets and the role of regulatory subsets including Tregs, MDSCs, tolerogenic DCs and mesenchymal stem cells (MSCs) (as summarised below and in Tables 2–4).

**Table 2:** T cells involved in graft-versus-host disease

Host (pre-conditioning)	No. of PBMCs (route)	Regime (route)	Clinical outcomes	Immunological outcomes^*^	Ref.
***Effector T cells***					
NSG	2 × 10^7^ (i.p.)	33 mg/kg cyclophosphamide days 3 and 4 (i.p.)	↓ Clinical score ↓ Weight loss ↑ Survival	↓ Tissue damage in liver ↓ Tregs	[79]
NOG (2 Gy)	5 × 10^6^ (i.v.)	50 mg/kg cyclophosphamide days 3 and 4 (i.p.)	- Weight loss ↑ Survival	- CD45^+^ ↓ CD3^+^ ↓ CD4^+^ ↓ CD8^+^ - Tregs ↓ B cells ↓ IFN-γ ↓ CD45^+^ in liver and lung	[89]
NSG	2 × 10^7^ (i.v.)	5 mg/kg 5-azacytidine every second day from days 1 to 21 (i.v.)	↓ Clinical score ↑ Survival	↓ Tissue damage in liver and lung ↑ Tregs ↓ IFN-γ ↓ TNF-α ↑ IL-2	[96]
NOG (2 Gy)	5 × 10^6^ (i.v.)	5 mg/kg anti-thymocyte globulin days -4 and -3 (i.p.)	↓ Weight loss ↑ Survival	↓ CD45 (prevented human cell engraftment)	[89]
NOG (2 Gy)	5 × 10^6^ (i.v.)	0.25 mg/kg alemtuzumab on days -4 and -3 (i.p.)	↓ Weight loss ↑ Survival	- CD45^+^ ↓ CD3^+^ ↓ CD4^+^ ↓ CD8^+^ ↓ Tregs ↓ B cells - IFN-γ - CD45^+^ in liver and lung	[89]
NSG (3 Gy)	1 × 10^7^ (i.p.)	5 μg OKT3 (anti-CD3) days 1, 3 and 5 (i.p.)	↑ Survival	↓ IFN-γ (day 7, 14 and 21) ↓ IL-2 (day 14) ↓ IL-8 (day 14) ↓ IL-10 (day 14 and 21)	[98]
NSG (3 Gy)	1 × 10^7^ (i.p.)	0.375 mg CsA daily from days 0 to 23 (i.p.)	↑ Survival	↓ IFN-γ (day 7 and 14) ↓ IL-2 (day 14) ↓ IL-8 (day 14) ↓ IL-15 (day 14) ↓ IL-17 (day 14)	[98]
NSG (3 Gy)	3 × 10^7^ (i.p.)	250 μg ILT3-Fc (anti-CD4) (i.p.) for 10 consecutive days and biweekly thereafter	↓ Clinical score ↑ Survival	↓ Leukocyte infiltration in liver and lung ↓ IFN-γ ↓ IL-5	[97]
NSG	2 × 10^7^ s (i.p.)	200 μg IT1208 (anti-CD4) twice weekly for 4 weeks (i.p.)	↓ Weight loss ↑ Survival	↓ Tissue damage in liver, skin and lung	[99]
NSG (2 Gy)	1.5 × 10^7^ (i.v.)	0.8 mg/kg anti-CD45RC mAb days 0 to 20 and every 2.5 days (i.p.)	↓ Weight loss ↑ Survival	Not reported	[100]
NSG	2.5 × 10^7^ (route not reported)	1 × 10^6^ CD83-targeted CAR T cells injected with PBMCs	↓ Clinical score ↑ Survival	↓ Tissue damage in liver and lung ↓ Th1 cells ↓ Th2 cells ↓ CD1c^+^CD83^+^ DCs ↓ CD1c^+^MHCII^+^ DCs	[101]
NSG	1 × 10^6^ (i.v.)	1 × 10^6^ Th17 cells co-injected with PBMCs	↑ Clinical score ↑ Weight loss ↓ Survival	↓ Tregs ↓ IFN-γ ↑ IL-17A	[102]
***Non-specific lymphocyte modulation***					
NSG (3 Gy)	1 × 10^7^ (i.p.)	50 mg gamunex once a week from days −1 to 42 (i.p.)	↑ Survival	↓ IFN-γ (day 7) ↓ IL-2 (day 14) ↓ IL-15 (day 14) ↓ IL-17 (day 7, 14 and 21)	[98]
NSG	1 × 10^7^ (i.p.)	50 mg gamunex once a week from days −1 to 42 (i.p.)	↓ Clinical score ↑ Survival	↑ NK cell expansion	[103]

Abbreviations: ↑, increased; ↓, decreased; -, no difference; CAR, chimeric antigen receptor; CD3^+^, T cells; CD4^+^, CD4^+^ T cells; CD8^+^, CD8^+^ T cells; CD45^+^, leukocytes; CsA, cyclosporin; GVHD, graft-versus-host disease; i.v., intravenous; i.p., intraperitoneal; NOG, NOD.*Cg-Prkdc^scid^Il2rg^tm1Sug^*; NSG, NOD.Cg-*Prkdc^scid^Il2rg^tm1Wjl^*; PBMC, peripheral blood mononuclear cell; Th1 cells, CD4^+^IFNγ^+^ T helper cells; Th2 cells, CD4^+^IL-4^+^ T helper cells; Tregs, regulatory T cells; IFN, interferon; IL, interleukin; ILT3, immunoglobulin-like transcript 3; mAb, monoclonal antibody; MHCII, major histocompatibility complex class II; NK, natural killer; TGF, transforming growth factor; TNF, tumour necrosis factor.

* All cells and molecules human unless stated as mouse.

**Table 3:** Regulatory T cells and myeloid derived suppressor cells in graft-versus-host disease

Host (pre-conditioning)	No. of PBMCs (route)	Regime	Clinical outcomes	Immunological outcomes^*^	Ref.
***Regulatory T cells***					
NSG	3 × 10^7^ (route not reported)	Co-transfer of 3 × 10^7^ rapamycin/TGF-β iTregs with PBMCs (1:8)	↑ Survival	↓ CD4^+^ ↓ CD8^+^	[115]
NSG (1.5 Gy)	1 × 10^7^ (route not reported)	Co-transfer of 1 × 10^7^ expanded thymic Tregs with PBMCs	↓ Clinical score ↓ Weight loss ↑ Survival	↓ Leukocyte infiltration in liver, ear and lung ↓ IFN-γ ↓ IL-6 ↓ IL-10	[112]
NSG (1.5 Gy)	1.5 × 10^7^ HLA-A^*^ 02^+^ (i.v.)	Co-transfer of MHC Class I 5-15 × 10^6^ A2-CAR CD8^+^ Tregs with PBMCs (1:1 or 3:1)	1:1 ratio: ↓ Weight loss ↑ Survival 3:1 ratio: ↑ Weight loss - Survival	↓ Leukocytes in blood ↓ Leukocyte infiltration in the liver and lung (both ratios, 1:1 better)	[116]
NSG (1.5 Gy)	1.5 × 10^7^ (i.v.)	Co-transfer of IL-34-expanded CD8^+^ Tregs with PBMCs (1:2)	↓ Weight loss ↑ Survival	Not reported	[117]
NSG (1.5 Gy)	1.5 × 10^7^ (i.v.)	0.4 mg/kg/2.5 d IL-34 and 0.8 mg/kg/2.5 anti-CD45RC mAb for 20 days	↓ Weight loss ↑ Survival	Not reported	[117]
NSG (1.5 Gy)	1.5 × 10^7^ (i.v.)	Co-transfer of expanded CD8^+^CD45RC^low/−^ Tregs with PBMCs (1:1 or 1:2)	↓ Weight loss ↑ Survival	Not reported	[115]
NSG (2 Gy)	1 × 10^7^ (i.v.)	1 × 10^6^ IL-27-stimulated Tregs (i.v.) day 0	↓ Clinical score ↑ Survival	↓ IFN-γ ↓ IL-6	[113]
NSG (3 Gy)	1 × 10^7^ (i.v.)	1 × 10^7^ cord blood derived Tregs day −1	↓ Clinical score ↓ Weight loss ↑ Survival	↓ IFN-γ ↓ TNF-α ↓ IL-5 ↓ IL-6	[114]
NSG (3.2 Gy)	1 × 10^7^ (i.v.)	1 × 10^7^ fucosylated Tregs day −1	↓ Clinical score ↓ Weight loss ↑ Survival	Not reported	[159]
NSG (2.5 Gy)	5 × 10^6^ (i.v.)	1 × 10^7^ TCR‐αβ^+^ double negative Tregs days 0, 3 and 7	↓ Weight loss ↑ Survival	Not reported	[160]
NOG (2 Gy)	2 × 10^7^ (i.v.)	5 × 10^6^ CD39^hi^ Tregs	↓ Weight loss ↑ Survival	↓ CD3^+^ in blood ↓ splenic IL-17 mRNA ↑ FoxP3	[111]
NOG (2 Gy)	2 × 10^7^ (i.v.)	5 × 10^6^ CD39^lo^ Tregs	- Weight loss - Survival	CD3^+^ in blood same as control ↑ Splenic IL-17 mRNA ↓ FoxP3	[111]
NOG (2 Gy)	2 × 10^7^ (i.v.)	5 × 10^6^ CD39^lo^ Tregs + IL-2	↓ Weight loss ↑ Survival	↓ Splenic IL-17 mRNA ↓ IFN- γ ↓ IL-6 ↓ IL-17	[111]
***Myeloid-derived suppressor cells***					
NSG	2 × 10^7^ (i.v.)	5 × 10^6^ human MDSCs co-injected with PBMCs (i.v.)	↓ Clinical score ↓ Weight loss ↑ Survival	↓ Tissue damage in liver ↑ FOXP3^+^ CD4^+^ and CD8^+^ cells	[119]
NSG (2 Gy)	1 × 10^6^ (i.v.)	1 × 10^6^ MDSCs (i.v.) days 21 and 24	↓ Clinical score ↓ Weight loss ↑ Survival	↑ Tregs ↓ IFN-γ^+^ cells ↓ TNF-α ↑ TGF-β ↓ IL-6 ↑ IL-10 ↓ IL-17	[120]
***Tolerogenic dendritic cells***					
NSG (1.5 Gy)	3 × 10^6^ (i.v.)	0.3 × 10^6^ autologous 1,25D_3_-DCs (i.p.)	Delayed GVHD onset	↑ Tregs	[121]

Abbreviations: ↑, increased; ↓, decreased; -, no difference; CAR, chimeric antigen receptor; CD3^+^, T cells; CD4^+^, CD4^+^ T cells; CD8^+^, CD8^+^ T cells; CD45^+^, leukocytes; DC, dendritic cell; GVHD, graft-versus-host disease; HLA, human leukocyte antigen; IFN, interferon; IL, interleukin; i.p., intraperitoneal; i.v., intravenous; iTregs, induced regulatory T cells; mAb, monoclonal antibody; MDSC, myeloid-derived suppressor cell; NOG, NOD.*Cg-Prkdc^scid^Il2rg^tm1Sug^*; NSG, NOD.Cg-*Prkdc^scid^Il2rg^tm1Wjl^*; PBMC, peripheral blood mononuclear cell; TGF, transforming growth factor; TNF, tumour necrosis factor; Tregs, regulatory T cells.

* All cells and molecules human unless stated as mouse.

**Table 4:** Mesenchymal stem cells in graft-versus-host disease

Host (pre-conditioning)	No. of PBMCs (route)	Regime (route)	Clinical outcomes	Immunological outcomes*	Ref.
NSG (3 Gy)	1 × 10^7^ (i.p.)	1 × 10^6^ cord blood derived MSCs (i.v.) day 0	↓ Clinical score ↓ Weight loss ↑ Survival	↓ Tissue damage in liver ↓ Engraftment (days 7 and 14)	[126]
NSG (2 Gy)	1 × 10^6^ (i.v.)	5 × 10^5^ cord blood derived MSCs (i.v.) days 0, 3 and 6	↓ Clinical score ↑ Survival	↓ Tissue damage and lymphocyte infiltration in liver, lung and intestine ↓ IFN-γ ↑ TGF-β ↑ PGE_2_	[123]
NSG-HLA-A2/HHD (2 Gy)	1 or 1.5 × 10^6^ (i.v.)	1–2 × 10^6^ cord blood derived MSCs (i.v.) on days 14, 18 and 22	- Clinical score - Survival	-Tregs - IL-10^+^ Tcons - IL-10^+^ CD8^+^ - IFN-γ^+^ Tcons - IFN-γ^+^ CD8^+^ - IL-17^+^Tcons	[124]
NOG	3 × 10^6^ (i.v.)	8 × 10^6^ cells/kg amnion derived MSCs (i.v.) administered once weekly for 3–4 weeks	↑ Survival	↓ Histological GVHD in the colon ↑ CD4^+^ ↓ CD8^+^ ↓ TNF-α ↓ PD-1^+^ CD8^+^	[125]
NSG-HLA-A2/HHD (2 Gy)	1 or 1.5 × 10^6^ (i.v.)	1–2 × 10^6^ bone marrow derived MSCs (i.v.) on days 14, 18 and 22	- Clinical score - Survival	- Tregs ↑ IL-10^+^ Tcons ↑ IL-10^+^ CD8^+^ - IFN-γ^+^ Tcons ↑ IFN-γ^+^ CD8^+^ - IL-17^+^Tcons	[124]
NSG	2 × 10^7^ (i.v.)	6 × 10^5^ to 6 × 10^6^ dental pulp derived MSCs (i.v.) at onset of GVHD symptoms	- Survival - Time to disease onset	- CD45^+^ cells or Tregs - IFN-γ - IL-17A - IL-10	[133]
NSG-HLA-A2/HHD (2 Gy)	1 or 1.5 × 10^6^ (i.v.)	adipose tissue derived MSCs (i.v.) on days 14, 18 and 22	- Clinical score - Survival	Not reported	[124]
NSG (2.4 Gy)	6.3 × 10^5^ g^−1^ (i.v.)	4.4 × 10^4^ g^−1^ MSCs on day 7 (i.v.)	- Clinical score - Weight loss ↑ Survival	↓ Histological GVHD in the liver and intestine - CD45^+^ - CD4:CD8 ratio - IL-2	[135]
NSG (2 Gy)	2.5 × 10^6^ (i.v.)	5 × 10^5^ small MSCs primed with hypoxia and Ca^2+^ (i.v.) day 18	↓ Weight loss ↑ Survival	↓ Tissue damage in liver, lung, kidney and intestine ↓ IFN-γ ↓ TNF-α ↓ IL-2	[136]
NSG (2 Gy)	1 × 10^7^ (i.v.)	2 × 10^6^ Apceth-201 MSCs (i.v.) days 14 and 18	↑ Survival	↓ IFN-γ ↓ TNF-α ↓ IL-5	[137]
NSG (2.4 Gy)	6.3 × 10^5^ g^−1^ (i.v.)	4.4 × 10^4^ g^−1^ IFN-γ stimulated MSCs on day 0	↓ Clinical score ↓ Weight loss ↑ Survival	↓ Histological GVHD in the liver and intestine - CD45^+^ - CD4:CD8 ratio - IL-2	[135]
NSG (2.5 Gy)	3 × 10^7^ (i.p.)	3 × 10^6^ MSCs (i.p.) days 0, 7, 14 and 21 and sirolimus 1.5 mg/kg day 0 to day 7 and 1 mg/kg (i.p.) days 8 to 21	↑ Survival	↓ IFN-γ ↓ IL-2 ↑ IL-6 ↓ IL-8 ↓ IL-10	[88]

Abbreviations: ↑, increased; ↓, decreased; -, no difference; CD3^+^, T cells; CD4^+^, CD4^+^ T cells; CD8^+^, CD8^+^ T cells; CD45^+^, leukocytes; GVHD, graft-versus-host disease; HLA, human leukocyte antigen; IFN, interferon; IL, interleukin; i.p., intraperitoneal; i.v., intravenous; MSC, mesenchymal stem cell; NOG, NOD.*Cg-Prkdc^scid^Il2rg^tm1Sug^*; NSG, NOD.Cg-*Prkdc^scid^Il2rg^tm1Wjl^*; PBMC, peripheral blood mononuclear cell; PGE_2_, Prostaglandin E_2_; Tcons, conventional CD4^+^ T cells; TGF, transforming growth factor; TNF, tumour necrosis factor; Tregs, regulatory T cells.

Tissue source of MSCs unknown in studies where not stated.

* All cells and molecules human unless stated as mouse.

### Effector T cells

CD4^+^ and CD8^+^ T cell subsets readily engraft in immune deficient mice [73–75,82–89], with an increased ratio of human CD4^+^:CD8^+^ T cells indicative of GVHD severity [90], reflecting observations in allogeneic HSCT recipients [91,92]. Injection of isolated human T-cell subsets into NOG mice has revealed both CD4^+^ and CD8^+^ T cells alone can mediate GVHD [93]. This same study revealed that γδ TCR-bearing T cells alone could not induce GVHD in this model, but whether they contribute to the individual effects of αβ TCR-bearing CD4^+^ or CD8^+^ T cells, or whether GVHD entirely relies on αβ TCR-bearing T cells remains to be determined. In contrast, another study, which also injected isolated human T-cell subsets into NOG mice, revealed that CD4^+^ but not CD8^+^ T cells alone could induce GVHD, with the latter requiring co-injection of at least half the number of CD4^+^ T cells to that of CD8^+^ T cells to induce GVHD [94]. Reasons for this discrepancy between the two studies may relate to five times the number of cells being injected in the former study compared with the latter. Notably, use of gene deficient bone marrow chimeric mice demonstrated that host APCs expressing MHC class II were required to induce GVHD suggesting that these murine cells could activate human CD4^+^ T cells [93]. Whether a similar role exists for host APCs expressing MHC class I and CD8^+^ T-cell activation was not assessed. However, earlier studies indicated that human CD4^+^ and CD8^+^ T cells proliferate in response to NSG mouse spleens and that this *in vitro* response and the development of GVHD *in vivo* involves the presence of MHC class II β chain and MHC class I β2 microglobulin chain, respectively [73,95], supporting a role for host murine APCs in the activation of donor human T cells.

Various studies have used small molecule inhibitors or biologics to study the role of T cells in humanised mice (Table 2). Use of anti-proliferative agents such as cyclophosphamide, azacytidine, anti-thymocyte globulin, alemtezumab [anti-CD52 monoclonal antibody (mAb)], OKT3 (anti-CD3 mAb), CsA, immunoglobulin-like transcript 3 (ILT3)-Fc, IT1208 (anti-CD4 mAb) and anti-CD45RC mAb to reduce GVHD in these models has highlighted the importance of T-cell proliferation in disease progression [79,89,96–100]. Moreover, co-administration of human CD83-targeted T cells into humanised mice demonstrated a role for activated CD83^+^CD4^+^ T cells including Th1 and Th2 cells but not Th17 cells, in GVHD progression [101]. However, a role for Th17 cells in GVHD progression cannot be excluded as others have shown that co-injection of human Th17 cells can worsen GVHD [102]. Notably, use of CD83-targeted T cells in humanised mice also revealed a role for human CD1c^+^CD83^+^ and CD1c^+^MHC class II^+^ DCs in GVHD progression, providing the first evidence that donor human DCs can contribute to GVHD development in humanised mice [101]. However, other evidence in this study indicated elimination of these DCs did not prevent GVHD entirely, suggesting that donor T cells are activated by other mechanisms.

Use of i.v. immunoglobulin, Gamunex, limited GVHD progression in humanised mice to a similar efficacy of CsA [98]. However, this improvement was not associated with impaired human T-cell proliferation, but rather immunomodulation, inhibiting secretion of T cell pro-inflammatory cytokines and promoting T cell dependent NK cell proliferation [103].

### Regulatory T cells

Tregs have firmly established roles in limiting or preventing GVHD development and progression [104–108]. In humans, the use of Tregs as a therapeutic strategy was initially limited by attempts to expand sufficient numbers of Tregs *ex vivo* for transfer, but strategies have now been developed to overcome this [46]. Current limitations relate to the persistence of Tregs and their suppressive capacity over time following transfer [46]. Therefore, strategies to expand Tregs *in vivo*, for example using low dose IL-2, have been examined and shown to increase Tregs to reduce GVHD development [109]. However, further studies into mechanisms for Treg expansion and stability of their suppressive function following transfer are required to optimise these approaches.

In NSG mice injected with hPBMCs, Tregs are known to be reduced over time [110]. Therefore, studies that investigate Tregs directly in humanised mice have largely relied on the co-administration of stimulated or expanded human Tregs with hPBMCs (Table 3). The majority of these studies show that the presence of donor human Tregs can reduce clinical GVHD in humanised mice. However, one study demonstrated that CD39^lo^ Tregs could not alter GVHD, but this effect could be overcome by the addition of IL-2 [111], indicating a central role for this cytokine in the promotion of donor Tregs to prevent GVHD. Evidence from a number of studies investigating Tregs in humanised mice suggested that impaired GVHD development was associated with reductions in human IFN-γ, TNF-α and IL-6 [111–114], indirectly supporting a role for these pro-inflammatory cytokines from donor cells in promoting GVHD. Notably, the effect of Tregs is not limited to CD4^+^ Tregs, with co-administration of CD8^+^ Tregs also able to reduce GVHD [115–117]. Finally, injection of low dose human IL-34 with depletion of CD45RC^high^ effector T cells reduced GVHD, corresponding to an increase in both CD4^+^ and CD8^+^ Tregs [108]. This effect of IL-34 was shown to be potently mediated by direct stimulation of Tregs and indirect stimulation of Tregs via monocytes. It will be of future interest to determine whether the recently described population of human CD8^+^ Tregs (KIR^+^CD8^+^ T cells), which can impair autoimmune disease [118], can also impact GVHD.

### Myeloid-derived suppressor cells and tolerogenic dendritic cells

MDSCs are a heterogeneous population of myeloid precursors with emerging inhibitory roles in preventing GVHD [45]. As for Tregs, human MDSCs have been investigated directly by the co-administration of these cells with hPBMCs (Table 3). In the two studies reported to date, co-administration of human MDSCs reduced clinical GVHD [119,120]. This reduction in GVHD was associated with increased human Tregs [119,120] and a corresponding reduction in human IFN-γ, TNF-α, IL-6 and IL-17 and increase in human TGF-β and IL-10 [120].

Finally, tolerogenic DCs may also have a role in preventing GVHD. Co-injection of human tolerogenic DCs (induced by active vitamin D) with hPBMCs reduced GVHD and corresponded to an increase in human Tregs [121]. Collectively, these studies support a role for both MDSCs and tolerogenic DCs in preventing GVHD development and progression, with their main mechanisms of action related to Treg development. However, a role for host MDSCs or tolerogenic DCs in preventing GVHD development in humanised mice remains to be determined.

### Mesenchymal stem cells

MSCs are multipotent stem cells that can not only differentiate into various cell types but can also regulate the immune response in many diseases including GVHD [122]. As such, a number of studies have revealed that the co-administration of hPBMCs with human MSCs, depending on the tissue source, can reduce GVHD (Table 4). MSCs derived from cord blood or the amnion appear most effective in preventing GVHD development in humanised mice [123–126], but their relevance in restricting GVHD in allogeneic HSCT recipients remains unclear.

Numerous clinical trials investigating MSCs as a therapy for GVHD in allogeneic HSCT recipients have been conducted, with the vast majority electing to use allogeneic bone marrow-derived MSCs [127–132]. These trials have shown promising overall responses but have a high transplant-related mortality rate with overall survival rates between 23 and 69%. In contrast, MSCs derived from adult bone marrow [124], as well as from dental pulp [133] or adipose tissue [124] have limited impact on GVHD in humanised mice which questions whether MSCs play a role in GVHD progression. However, a recent systematic review of the literature revealed that infusion of MSCs in allogeneic HSCT recipients can reduce the development of chronic but not acute GVHD [134]. Thus, providing an explanation for the lack of efficacy of certain MSCs in humanised mice (Table 4), which largely develop acute GVHD. Moreover, in addition to the various tissue sources of MSCs used in humanised mouse studies, studies reported differing immunological outcomes making a comparison between studies difficult. Additional research is needed to further elucidate the role of MSCs in GVHD, as well as their potential as a cellular therapy in the prevention of this disease. To this end, some studies with humanised mice have used *ex vivo* manipulated MSCs prior to injection, reporting decreased GVHD [135–137].

## Cell signalling mechanisms in GVHD

Inhibitors and ligands have been examined in a number of humanised mouse studies and have helped elucidate cell signalling mechanisms in GVHD. The majority of such studies have shown the prevention or reduction of GVHD following administration of these compounds, highlighting important roles for DAMPs (and their receptors), co-stimulatory molecules, cytokines (and their receptors) and intracellular pathways in GVHD development (as summarised below and in Table 5).

**Table 5:** Cell signalling mechanisms in graft-versus-host disease

Host (pre-conditioning)	No. of PBMCs (route)	Target	Regime (route)	Clinical outcomes	Immunological outcomes^*^	Ref.
***DAMP signals***						
NSG	1 × 10^7^ (i.p.)	CD39/CD73	50 mg/kg αβ-methylene-ADP days 0–6 (i.p.)	↑ Weight loss	↑ Leukocyte infiltration in liver ↑ IL-2 ↓ CD4^+^CD39^−^CD73^−^ T cells in blood	[138]
NSG	1 × 10^7^ (i.p.)	P2X7	50 mg/kg BBG days 0, 2, 4, 6, 8 and 10 (i.p.)	- Weight loss - Clinical score - Survival	↓ Tissue damage in liver, skin and intestine ↓ IFN-γ	[84]
NSG	1 × 10^7^ (i.p.)	P2X7	50 mg/kg BBG thrice weekly until endpoint (i.p.)	- Weight loss - Clinical score - Survival	↓ Tissue damage in liver	[85]
NSG	1 × 10^7^ (i.p.)	P2X7	50 mg/kg BBG days 0 to 10 (i.p.)	↓ Clinical score	↓ Tissue damage in liver, skin, ear and lung ↑ Tregs ↑ B cells ↓ IFN-γ	[80]
NSG	1 × 10^7^ (i.p.)	P2X7	300 mg/kg PPADS days 0 to 10 (i.p.)	↓ Ear thickness	↑ Tregs	[80]
NSG	1 × 10^7^ (i.p.)	A_2A_	0.1 mg/kg CGS 21680 days −2 to 11 (i.p.)	- Weight loss - Clinical score - Survival	↓ Tissue damage in liver ↓ Tregs ↓ TNF-α ↑ IL-6	[140]
***Co-stimulatory molecules***						
NOG	1 × 10^7^ (i.p.)	CD26	200 μg/dose CD26 mAb thrice weekly for 10 doses (i.p.)	↓ Clinical score ↓ Weight loss ↑ Survival	↓ Tissue damage in liver ↓ CD26^+^ cells in blood ↓ Proliferation of CD8^+^ T cells in spleen ↓ IFN-γ in the liver ↓ TNF-α in the liver ↓ IL-2 (mRNA expression) ↓ IL-4 in the liver ↓ IL-17A in the liver	[141]
NOG	1 × 10^7^ (i.p.)	CD28	200 μg/dose CTLA4-Ig thrice weekly for 10 doses (i.p.)	↓ Clinical score ↓ Weight loss ↑ Survival	↓ IFN-γ ↓ TNF-α ↓ IL-2 ↓ IL-4 ↓ IL-17A	[141]
NSG (1.8 Gy)	1× 10^7^ (i.v.)	CD38	5 mg/kg daratumumab day 0, 7 and 14 (i.p.)	↓ Clinical score ↓ Weight loss ↑ Survival	↓ Tissue damage in liver and lung ↓ CD45^+^ ↓ CD3^+^ ↑ CD4^+^ ↓ CD8^+^ ↓ CD4^+^:CD8^+^ ratio ↓ Ki67^+^ CD4^+^ ↓ Ki67^+^ CD8^+^ ↓ CD69^+^ CD8^+^ ↓ CD45RA^+^CCR7^+^ CD4^+^ ↓ CD45RA^+^CCR7^−^ CD4^+^ ↑ CD45RA^+^CCR7^+^ CD8^+^ ↑ CD45RA^−^CCR7^+^ CD8^+^ ↓ CD45RA^−^CCR7^−^ CD8^+^ ↑ Tregs	[142]
NSG (2 Gy)	2 × 10^6^ (r.o.)	ICOS	500 µg anti-ICOS mAb (i.p.) day 0	↓ Weight loss ↑ Survival	↓ Leukocyte infiltration in liver and lung	[143]
NSG (2.5 Gy)	5 × 10^6^ (i.v.)	TIM-1	250 μg Anti-TIM-1 mAb days −1, 3, 7 and 11 (i.p.)	↑ Survival	Not reported	[144]
NSG	1 × 10^7^ (route not reported)	PD-1	10 mg/kg sasanlimab days 2 and 8	↑ Weight loss ↓ Survival	↑ IFN-γ	[145]
***Cytokine and chemokine signals***						
NSG (2 Gy)	2 × 10^7^ (i.v.)	TNF-α	100 µg etanercept days −3 and −1 and every 3 days after PBMCs (i.v.)	↓ Weight loss ↑ Survival	↓ CD45^+^ cells	[73]
NSG	3 × 10^7^ (i.v.)	IL-21	200 μg anti-IL-21 mAb day −1, then 3 times weekly until day 41 (i.p.)	↓ Weight loss ↑ Survival	↑ Tregs ↓ IFN-γ^+^ T cells ↓ IL-17^+^ T cells	[147]
NSG	2 × 10^6^ (i.v.)	IL-2 inhibition through IL-10	50 µg of plasmid expressing IL-2 and IL-10 on day 0 (i.p.)	↑ Survival	↓ CD45^+^ cells ↑ CD4^+^ T cells	[148]
NSG (IL-10 expressing)	1 × 10^7^ (i.v.)	IL-10	100 µg anti-IL-10 mAb day 6 then twice weekly for five injections (i.v.)	↓ Weight loss ↑ Survival	↓ Tissue damage in liver	[149]
NSG (hIL-10 expressing)	1 × 10^7^ (i.v.)	-	-	↑ Weight loss ↓ Survival	↑ Tissue damage in liver ↑ TNF-α ↓ IL-2^+^ cells ↑ IL-10	[149]
NOG-hIL-4-Tg (2.5 Gy)	2.5 – 5 × 10^6^ (i.v.)	IL-4 treatment		↑ Survival	↓ CD45^+^ ↓ CD3^+^	[150]
NOG	5 × 10^7^ (i.p.)	IL-18 treatment	15 μg SB-485232 daily for 3 weeks subcutaneous	↓ Survival	↑ CD45^+^ cells ↑ CD8^+^ cells ↓ Tregs	[151]
NSG (2.2 Gy)	7 × 10^6^ (route not reported)	CCR7	1 mg R707 day 0, then 500 μg days 1, 2, 5, 8, 12, and 15 (i.p.)	↓ Clinical score ↑ Survival	↓ Leukocyte infiltration in spleen	[152]
***Intracellular signaling molecules***						
NSG	2 × 10^7^ (i.v.)	JAK 1	3 mg itacitinib twice daily from days 3 to 28 (orally)	↓ Clinical score ↓ Weight loss ↑ Survival	↑ Tregs (day 21 and 28) ↓ CD4^+^ T cells ↓ CD8^+^ T cells ↓ IFN-γ ↓ TNF-α ↑ IL-2	[153]
NSG	3 × 10^7^ (i.p.)	Aurora kinase A and JAK 2	30 mg/kg alisertib daily and TG101348 (45 mg/kg) twice daily to day 14 (i.p.)	↓ Clinical score ↑ Survival	Not reported	[154]
NSG	3 × 10^7^ (i.p.)	Aurora kinase A and JAK 2	50 mg/kg AJI-100 daily to day 14 (i.p.)	↓ Clinical score ↑ Survival	↓ Tissue damage in liver and lung ↑ Treg: Tcon ratio ↓ CD4^+^ T cells ↓ Th17 ↓ Th1	[154]
NSG	2.5 × 10^7^ (i.p.)	JAK 2 and STAT3	0.5 mg/kg sirolimus with 2.5 mg/kg of S3I-201 days 0 to 14 (i.p.)	↓ Clinical score ↑ Survival	↑ Tregs ↓ IFN-γ^+^ cells ↓ Th1^+^ cells ↓ Th17 cells	[155]
NSG (2.5 Gy)	2 × 10^6^ (i.v.)	mTOR	1 mg/kg rapamycin daily from days 1 to 21 (i.p.)	↓ Clinical score ↑ Survival	↑ Tregs ↓ IFN-γ ↓ TNF-α ↑ IL-2 ↓ IL-17	[156]
NSG (2.5 Gy)	5 × 10^6^ (route not reported)	Glycogen synthase kinase 3	30 mg/kg 3, 6-bromoindirubin 3′-oxime day 0 to 3 and 3 mg/kg day 5 to 8, 10 to 13, and 15 to 17 (i.p.)	↓ Weight loss ↑ Survival	↓ Tissue damage in liver	[157]

Abbreviations: ↑, increased; ↓, decreased; ADP, adenosine 5′-diphosphate; BBG, Brilliant Blue G; CCR7, C-C chemokine receptor type 7; CD3^+^, T cells; CD4^+^, CD4^+^ T cells; CD8^+^, CD8^+^ T cells; CD45^+^, leukocytes; CTLA4, cytotoxic T lymphocyte antigen 4; DAMP, damage-associated molecular pattern; GVHD, graft-versus-host disease; IFN-γ, interferon gamma; ICOS, inducible co-stimulator; IL, interleukin; i.p., intraperitoneal; i.v., intravenous; JAK, Janus-associated kinase; mAb, monoclonal antibody; mTOR, mechanistic target of rapamycin; NOG, NOD.*Cg-Prkdc^scid^Il2rg^tm1Sug^*; NSG, NOD.Cg-*Prkdc^scid^Il2rg^tm1Wjl^*; PBMC, peripheral blood mononuclear cell; PPADS, pyridoxalphosphate-6-azophenyl-2',4'-disulfonic acid; r.o., retro-orbital; STAT3, signal transducer and activator of transcription 3; Tg, transgenic; TGF, transforming growth factor; Th, T-helper; TNF, tumour necrosis factor; Tregs, regulatory T cells.

* All cells and molecules human unless stated as mouse.

### Damage-associated molecular patterns and their receptors

DAMPs represent a diverse range of molecules that can be released in response to tissue injury or during cellular stress to act as danger signals to promote GVHD [2]. Studies of DAMPs in humanised mice are limited to extracellular ATP and activation of purinergic receptors. A role for extracellular ATP in humanised mice is indirectly evidenced by the blockade of CD39 (ectonucleoside triphosphate diphosphohydrolase-1) and CD73 (5′-nucleotidase), which increases extracellular ATP, and was shown to worsen GVHD [138]. Furthermore, blockade of the main ATP-gated receptor, P2X7, on immune cells reduced clinical and histological GVHD in humanised mice [80,84,85], which corresponded to a decrease in human IFN-γ and an increase in Tregs [80]. Notably, each of these studies used non-irradiated mouse models, indicating that ATP release is not limited to tissue damage following irradiation. This supports a role for both ATP release via other mechanisms and the ATP-P2X7 receptor signalling axis in GVHD progression.

Conversely, hydrolysis of extracellular ATP by CD39 and CD73 can lead to the generation of adenosine, which can bind A_2A_ receptors on immune cells to dampen GVHD [139]. Consistent with this, activation of the A_2A_ receptor in humanised mice resulted in a decrease in liver GVHD and human TNF-α [140]. However, this treatment also reduced donor human Tregs and increased human IL-6 confounding the precise role of the adenosine-A_2A_ receptor pathway in this model of GVHD. Thus, further research is required to understand this pathway in humanised mice and allogeneic HSCT recipients.

### Co-stimulatory molecules

Molecules involved in co-stimulation of T cells play important roles in inducing GVHD, with blockade of these molecules providing important insights into the mechanisms of GVHD. CD26, CD28 and CD38 are co-stimulatory molecules present on T cells with emerging roles in GVHD. Inhibition of CD26 by a neutralising mAb or CD28 by the inhibitory ligand cytotoxic T-lymphocyte-associated protein 4 (CTLA-4; CD152) reduced GVHD, which corresponded to a reduction in a number of human pro-inflammatory cytokines including IFN-γ, TNF-α, IL-2, IL-4 and IL-17A in humanised mice [141]. Inhibition of CD38 also resulted in reduced clinical and histological GVHD [142]. Likewise, blockade of inducible T cell co-stimulator (ICOS; CD278) and T cell immunoglobulin and mucin -1 (TIM-1; CD365), co-stimulatory molecules present on T cells, by neutralising mAbs can also reduce GVHD in humanised mice [143,144]. Collectively, these studies reveal roles for activation of each of these co-stimulatory molecules in the promotion of GVHD.

In contrast with the co-stimulatory molecules above, mAb blockade of the immune checkpoint molecule programmed cell death (PD) molecule-1 (PD-1; CD279) worsened GVHD in humanised mice, which corresponded to an increase in human IFN-γ [145]. This suggests that PD-1 and its ligands, PD-L1 and PD-L2, act to prevent GVHD development and that use of immune checkpoint blockade to prevent cancer in allogeneic HSCT recipients increases the risk of GVHD. Thus, as discussed elsewhere [146], use of immune checkpoint blockade may be limited for HSCT recipients following cancer relapse.

### Cytokines and their receptors

Cytokines, including chemokines, and their respective receptors play a pleiotropic role in cell signalling. These have been indirectly studied in the context of GVHD development and progression with a limited number of studies directly investigating cytokines and their receptors in humanised mice. The latter will be the focus below.

Blockade of cytokines or their receptors has revealed the role of signalling axes in the development of GVHD in humanised mice. Fusion protein blockade of the soluble TNF-α receptor delayed progression of GVHD, which corresponded to a reduction in human leukocyte engraftment and was interpreted to be an indicator of GVHD development [73]. Blockade of human IL-21 by a mAb also reduced GVHD development, which resulted in a reduction in both human Th1 and Th17 cells, as well as an increase in human Tregs [147]. Notably, IL-17 has been associated with skin GVHD. mAb blockade of IL-17 reduced skin GVHD in NOG mice engrafted with human CD4^+^ T cells, corresponding to a reduction in murine neutrophils and macrophages, and reduced C-X-C chemokine ligand (CXCL) 1 and CXCL5 in skin lesions [75]. Moreover, this same study, which as discussed above demonstrated that human CD8^+^ T cells alone could not engraft NOG mice, revealed that the transgenic expression of human IL-2 could support CD8^+^ T cell engraftment and GVHD development, with the authors suggesting that IL-2 from CD4^+^ T cells facilitates CD8^+^ T cell engraftment in this model following injection of hPBMCs. Consistent with this, transgene expression of human IL-2 promotes the expansion of both human CD4^+^ and CD8^+^ T cells and GVHD development in NSG mice [148]. However, IL-2 is also important in the establishment of Tregs and the prevention of GVHD in humanised mice [110], an effect possibly explained by enhancement of IL-10. Transgene expression of both human IL-2 and IL-10 preferentially promotes the expansion of human CD4^+^ T cells and limits GVHD development compared with transgene expression of IL-2 alone [148]. Paradoxically, transgene expression of human IL-10 promotes human T cell expansion and GVHD severity, which can be reduced by mAb blockade of IL-10 [149]. Collectively, these two studies suggest that in presence of IL-2, IL-10 has an anti-inflammatory role, possibly via promotion of Tregs, but in the absence of IL-2, IL-10 has a pro-inflammatory role in GVHD.

Two studies have examined the impact of exogenous cytokines on GVHD development in humanised mice. Transgene delivery of human IL-4 was shown to reduce GVHD, which corresponded with a reduction in human T-cell engraftment [150], suggesting a beneficial role for this cytokine in preventing GVHD development. Conversely, injection of human IL-18 increased GVHD, which corresponded to increased CD8^+^ T cells and decreased Tregs [151], possibly as a consequence of increased human IFN-γ but this cytokine was not investigated.

Finally, blockade of C-C chemokine receptor (CCR) 7 (CD197), which facilitates leukocyte trafficking into secondary lymphoid tissues, reduced GVHD in humanised mice, which corresponded to a reduction in leukocyte engraftment in spleens of these mice [152]. This study provides an example of a cytokine-cytokine receptor axis that can cross the species barrier in humanised mice and highlights the importance of leukocyte trafficking in GVHD development and progression. Together, the studies above highlight the important roles that interleukins and chemokines and their respective receptors play in the induction of GVHD.

### Intracellular signalling molecules

By extension, the above studies of DAMPs, co-stimulatory molecules and cytokines indirectly implicate a number of intracellular signalling pathways in the development and progression of GVHD. As such, numerous studies have used small molecule inhibitors to investigate the role of various intracellular signalling molecules in GVHD (and their potential as therapeutic targets). Blockade of JAK 1, which is downstream of IL-2, IL-4 and IL-6 receptor family members, demonstrates a role for this kinase in GVHD development. Blockade of JAK 1 corresponded to increased human CD4^+^ and CD8^+^ T cell engraftment and reduced human Treg engraftment [153]. Dual blockade of Aurora Kinase A and JAK 2, which are downstream of CD28 and IFN-γ, IL-6 and granulocyte-macrophage colony-stimulating factor (GM-CSF) receptor family members, respectively, impaired the development of human Th1 and Th17 cells and increased human Treg numbers [154]. Likewise, dual blockade of JAK 2 and signal transducer and activator of transcription 3 (STAT3), which is activated by JAK, corresponded to reduced human Th1, Th17 and IFN-γ ^+^ cells and increased human Tregs [155]. Collectively, these studies indicated that JAK activation holds a significant role in GVHD pathogenesis, but further studies are needed to confirm the specific role of each kinase in this process.

Other studies of humanised mice indicate that kinases unrelated to the above are also involved in GVHD progression. Blockade of mTOR has demonstrated a role for this kinase in the development of GVHD through production of human IFN-γ and TNF-α and impairment in human Treg numbers [156]. Finally, blockade of glycogen synthase kinase 3, which has important roles in the activity of T cells and other leukocytes, supports a role for this enzyme in the development of GVHD [157].

## Conclusion

It is evident that humanised mouse models are a useful preclinical tool to examine the mechanisms of GVHD development. Further studies investigating the specific role of CD4^+^ and CD8^+^ T cells in the pathogenesis of GVHD are required, as evidence from current studies is still conflicting. Furthermore, the role of Th17 cells and the mechanisms behind their pathogenicity in a GVHD setting, as well as other Th subsets need to be further explored. Whilst there are numerous studies that investigate the role of CD4^+^ Tregs in alleviating GVHD, future studies should seek to determine the efficacy of other regulatory cells such as KIR^+^CD8^+^ T cells and MDSCs. Further investigation of key points within cell signalling pathways may also elucidate additional mechanisms through which GVHD arises in humanised mice. Outcomes of these studies may provide valuable insights into the best approach to prevent and reduce GVHD and lead to better biomarkers for predicting disease development in a clinical setting. Finally, studies that investigate such mechanisms need to assess the role of these mechanisms in the development of GVT immunity to prevent increased rates of disease relapse.

## Abbreviations

α4β7: alpha 4 beta 7
ADP: adenosine 5′-diphosphate
APC: antigen-presenting cell
ATG: anti-T cell globulin
ATP: adenosine 5′-triphosphate
BRG: C.Cg-*Rag2^tm1Fwa^IL2rγ^tm1Sug^*
CAR: chimeric antigen receptor
CCR: C-C chemokine receptor
CsA: cyclosporin
CTLA4: cytotoxic T-lymphocyte associated protein 4
CXCL C-X-C: chemokine ligand
CXCR C-X-C: chemokine receptor
DAMP: damage-associated molecular pattern
DC: dendritic cell
GVHD: graft-versus-host disease
GVT: graft-versus-tumour
HLA: human leukocyte antigen
hPBMC: human peripheral blood mononuclear cell
HSC: haematopoietic stem cell
HSCT: haematopoietic stem cell transplantation
ICOS: inducible T-cell co-stimulator
IFN: interferon
IL: interleukin
ILT3: immunoglobulin-like transcript 3
IL-2R: interleukin-2 receptor
i.p.: intraperitoneal
i.v.: intravenous
JAK: Janus-associated kinase
KIR: killer cell immunoglobulin-like receptors
mAb: monoclonal antibody
MDSC: myeloid derived suppressor cell
MHC: major histocompatibility complex
miR: microRNA
MSC: mesenchymal stem cell
mTOR: mammalian target of rapamycin
NBSGW: NOD.Cg-*Kit^W-J^Tyr^+^Prkdc^scid^Il2rg^tm1Wjl^*
NK: natural killer
NOD: non-obese diabetic
NOG: NOD.Cg-*Prkdc^scid^Il2rg^tm1Sug^*
NPG: NOD.*Prkdc^scid^Il2rg^null^*
NSG: NOD.Cg-*Prkdc^scid^Il2rg^tm1Wjl^*
PAMP: pathogen-associated molecular pattern
PD: programmed cell death
PTCy: post-transplant cyclophosphamide
SIRPα: signal regulatory protein-α
STAT3: signal transducer and activator of transcription 3
TCR: T-cell receptor
TGF: transforming growth factor
Th: T helper
TIM-1: T-cell immunoglobulin and mucin-1
TNF: tumour necrosis factor
Treg: regulatory T cell
